# The Divine in the Clinic: Assisted Reproduction and Religious Practice in Ghana and South Africa

**DOI:** 10.1007/s10943-024-02222-1

**Published:** 2025-01-12

**Authors:** Andrea Whittaker, Trudie Gerrits, Lenore Manderson

**Affiliations:** 1 School of Social Sciences, 20 Chancellor’s Walk, Monash University, Melbourne, VIC 3880 Australia; 2 Anthropology Department, University of Amsterdam, Amsterdam, The Netherlands; 3 School of Public Health, University of the Witwatersrand, Johannesburg, South Africa

**Keywords:** Assisted reproductive technologies, South Africa, Ghana, Ritual, Christianity, Ancestors

## Abstract

Drawing on studies with 40 informants in Ghana and 74 informants in South Africa, we explore spiritual interventions among staff and patients that accompany their use of assisted reproduction. These practices and expressions of faith reinforce staff and patients as moral subjects who have done everything possible to assist in the vagaries of assisted reproduction—another form of care to enable, complement, and enhance high-tech intervention. We consider the creation of sacred spaces in the clinics, the rituals that form part of IVF practice, and the dilemmas of translation when assisted reproductive technologies (ARTs) travel to different cultural and religious contexts.

## Introduction

The unpredictability of conception invites appeals to interventions by gods, ancestors, and at times, assisted reproductive technologies (ARTs). Anthropological research has drawn attention to the manifold ways in which religious beliefs inform the legal environment and everyday life, so impacting the availability of and access to assist reproductive technologies and their actual use. Assisted reproductive technologies “are sociotechnical entities” (Inhorn & Birenbaum-Carmeli, 2008, p. 178); they are not transferred into cultural voids. Rather, local considerations and circumstances—cultural, social, religious, economic, legal, and political—shape and sometimes curtail the ways these techniques are offered and received in different contexts (Inhorn, 2003; Inhorn & Birenbaum-Carmeli, 2008).

Since the introduction in the 1980s of IVF to enable pregnancy under certain circumstances, religious institutions have grappled with questions of the ethics and morality of the use of these technologies; in observing this, a number of anthropologists have documented how formal religions navigate, resolve, or curtail these issues. For example, in Islam, Sunni Muslims are permitted to use IVF, but not to make use of egg and sperm donation and surrogacy, to avoid the sin of adultery; for Shi’ite Muslims, these latter forms of assisted reproduction are allowed under certain circumstances (Inhorn, 2006, 2015; Inhorn et al., 2017; Tremayne, 2009). In contrast, the state of Israel allows and subsidizes all forms of ARTs. This includes the use of donor gametes and surrogacy to reproduce Jewish babies; and orthodox rabbis are highly involved in designing clinical protocols to guarantee that the offspring is considered Jewish, even when gamete donation is involved (Ivry, 2009, 2010; Kahn, 2000).

The Roman Catholic Church is the only major world religion that has denounced all forms of assisted reproduction, as the use of ARTs implicates the elimination of embryos (that is of human life) and because, by using ARTs, “humans are technologically interfering with a process that should remain under God’s dominion” (Ratzinger, 1987 in Roberts, 2012: 507). Still, many Catholics make use of these technologies (see Gerrits, 2016 for the Netherlands).

The Greek Orthodox Church was also reluctant to support ART legislation, when the Greek government proposed the first Bill on “Medically Assisted Human Reproduction” in 2002 (Paxson, 2006). This bill intended to give legal status to ART practices that had developed in Greece over more than a decade, including post-mortem fertilization, anonymous gamete donation, and accessibility for lesbian couples. The Greek Orthodox Church opposed lesbian access to ARTs as it would give way for “the legalization of relationships and childbearing contrary to nature with destructive consequences upon the child and society” (Paxson, 2006, p. 498).

Another body of anthropological research has shown how, during treatment, individual patients and clinical staff draw on faith when navigating the uncertainties of assisted reproduction: “Religion frequently enters the lab and the clinic, through the words and actions of both patients and practitioners, in ways that makes treatment possible” (Thompson, 2006, p. 557). For example, in Thailand, infertility is viewed as a consequence of one’s past deeds in previous lives, karma (*kaam*) and fate (*khro*), as much as it is considered a medical issue. Couples experiencing fertility difficulties consequently implore a fertility pantheon for intervention—requesting, begging, and bargaining for spiritual interventions in their quest to form a family at the same time as they undertake assisted reproductive treatment (Whittaker, 2015).

In Indian IVF clinics, Aditya Bharadwaj (2006) found that doctors drew on “the idea of divine intervention in the process of clinical conception”, reflecting both firm religious convictions that help them to understand limited success rates and successes, and because “turning to God is … a way of managing the anxiety that typically accompanies the running of a successful IVF program” (2006, p. 456). Bharadwaj highlights the enormous pressure on clinic staff to ensure successful pregnancy, in order to be commercially feasible.

A study of IVF clinics in Ecuador by Elizabeth Roberts (2012) found that IVF practitioners disagreed with the Catholic Church’s characterization that they were “playing God”; rather, they saw themselves as “God’s helpers” (2012, p. 525). At crucial moments of fertilization and embryo transfer, practitioners performed “visible acts of devotion” and “silent prayers”, expressing their dependency upon and “humility before God” (2012, p. 518). Evoking God’s presence in the clinic and laboratory is a way to challenge the Church’s disapproval of IVF practice. Yet at the same time, although the Vatican taught that IVF involves the destruction of human life, for many practitioners and patients alike, embryos did “not necessarily signify life” (Roberts, 2012, p. 525); leftover embryos after an embryo transfer were often “unceremoniously” dumped in the trash (p. 517).

Little has been published on the role of religion with regard to ARTs in African contexts, notwithstanding the vital role of different religions on the continent and the everyday evidence of religiosity (e.g. Hiadzi et al., 2021). In Ghana, Kristine Krause (2012) refers to the importance of prayer over the medicines in IVF for Ghanaian Pentecostal Christians, as “a way of coping with the unpredictability of [their] efficacy” (2012, p. 227), but also reflecting the tendency among this group of Christians to “use anything available as point of contact to God’s healing power” (2012, p. 233).

In this article, we describe the important roles of various forms of spiritual beliefs within high-technology-assisted reproductive treatment in Ghana and South Africa; we explore both spiritual and religious interventions and faith-based concerns of staff and patients that accompany their medical interventions. We consider these practices and expressions of religiosity as part of a religious “heterotopia” (Taylor, 2008, p. 21 following Foucault), in which boundaries between the sacred and mundane blur in everyday life, and in which the imaginaries of a future family are inscribed. As we demonstrate, intending parents and the professionals who support them are confronted by the always uncertain outcomes of IVF, its medical risks and miscarriages, and the role of human intervention in creating potential new lives and kin. Faith, magico-religious thinking and rituals play a significant role, even among those committed professionally and personally to “high-tech” scientific interventions.

The study participants were all patients undertaking high-tech-assisted reproductive technologies to treat their infertility, or were medical staffs providing relevant care. For both groups, drawing on religious belief and appealing to the divine were not a last resort; it was, rather, a form of care and intervention which complemented and enhanced other interventions. Such acts reinforce the supplicants’ status as moral subjects, who are doing everything possible to ensure the success of treatment. We explore the creation of sacred spaces in clinics; the positioning of clinicians, embryologists, patients, and donors as moral subjects; the rituals undertaken that form part of IVF practice; and new dilemmas of translation when ARTs travel to different cultural and religious contexts.

## Methods

We draw on fieldwork data from two broader projects in two clinics in Ghana in 2012–2013 and work in South Africa in 2022 and 2023 which involved site visits to seven assisted reproduction clinics. In Ghana, Gerrits conducted observations in different spaces in two clinics, including consultation and treatment rooms, waiting rooms, the “baby room” and—to a limited extent—IVF laboratories; she held in-depth interviews and had informal conversations with 12 various staff members, 22 women and men in treatment, and 6 surrogates. In Ghana, fieldwork involved several week’s observations in the two clinics.

In South Africa in 2022 and 2023, Whittaker and Gerrits made seven site visits to five private and two public clinics across the country. At these sites, we undertook observations throughout the clinic and laboratories when possible and undertook in-depth interviews and informal conversations with staff. This usually involved visits ranging from several hours to one day, and included tours of facilities, interviews with staff, and the opportunity to see staff at work and sit unobtrusively in waiting rooms with patients. In this article, we draw upon interviews conducted in South Africa with 17 fertility specialists, 7 embryologists and 12 nurses/donor coordinators, 7 other allied staff, 18 other staff, and 13 egg donors and others (see Table 1).

**Table 1:** Participants from South Africa and Ghana (*n* = 114)

Occupation	South Africa	Ghana
Fertility specialists	17	4
Embryologists	7	3
Nurses/donor coordinators	12	5
Allied Health Staff	7	
Other staff^a^	18	
Egg Donors	13	
Patients		22
Surrogates		6
Total	**74**	**40**

^a^Included translators; clinic coordinators; travel facilitators; administrative staff

Interviews were undertaken by experienced anthropologists; in South Africa, some interviews were conducted by both the first and second authors together. Interviews averaged approximately one hour in length and were semi-structured, with open-ended questions used to elicit narratives from informants about their experience. Interviews covered a range of issues, but included open prompt questions asking informants to reflect upon their experiences. These were thematically coded and comparatively analysed; major themes are presented below. Religion was not the main topic of interviews, nor did it arise spontaneously in every interview; here, we focus on cases when it did arise as a topic of discussion.

Ethical clearance was granted by the Monash University (MUHREC 27166), the University of the Witwatersrand (M210546), and participating clinics. All participants gave informed signed consent to be interviewed. Ethical clearance for the Ghanaian Project was obtained through the Noguchi Memorial Institute for Medical Research-IRB in Accra, Ghana.

Due to the small community of staff involved in assisted reproduction in these countries, participant demographic data are not provided as this would impinge upon the anonymity of participants. Pseudonyms are used throughout for all informants and clinic names.

### Religion in Ghana and South Africa

In Sub-Saharan Africa, religious beliefs and practice include apostolic worship, ancestral rituals, divination and traditional healing, and African churches, combined with charismatic, Pentecostal and mainstream Christianity, Islam, Hinduism, and Judaism. These faiths and their various sects all place high value on women demonstrating their fertility and producing children.

Ghana is ranked among the 10% most strongly religious countries in the world. Some 72% of the population belong to a Christian denomination: 28.3% of the population are Pentecostal Protestants; 18.4% are non-Pentecostal Protestants; and 13.1% Catholic Christians. Islam is the second most common religion, and 17.6% of the population adhere to it (the majority of Muslims in Ghana are Sunni; 8% are Shia) (StatsGhana, 2023). African traditional religion continues to play a role for 5.2 % of the population, with “different supernatural beings—namely the Supreme Being, the nature gods, the ancestors, and the other gods”—all playing a role in individuals’ fertility (Hiadzi et al., 2021, p. 2). Religion is evident in the everyday life of its population and 94% of women report to be participants in churches and charismatic prayer circles (Gallup 2023; Hiadzi et al., 2021, p. 2–4). People include religious blessings in emails and biblical quotes on telephone contact numbers on smartphones. Bibles routinely sit on desks next to office files and diaries of forthcoming commitments. As “ancestors are believed to give children to the living for the continuation of the lineage”, believers pray to them for fertility (2021, p. 2). Certain deities are thought to “bless ‘good’ people with many off-spring”, and the inability to conceive is regarded as a sort of punishment (2021, p. 3). In addition, witchcraft is held responsible for part of the fertility problems.

In South Africa, freedom of religion is a tenet of the Constitution. Around 15% declare that they have no religious affiliation or declined to state one; some 5% are Muslim, Hindu, Jewish, Buddhist, or of other faiths, visible primarily in major cities; some 5% adhere to traditional African religions. The majority 80% of the population is Christian, including mainstream Protestant churches and the Catholic Church, Pentecostal churches, and apostolic, African Zionist^1^ (*amaZioni* in Zulu) and other African initiated churches. For most South Africans, although religion is “complex, diverse, and evolving” (Atabongwoung et al., 2023, p. 486), adherence to faith and religiosity is an integral aspect of everyday life: this includes prayer, reading religious texts, and attending religious services (Farrar et al., 2019). As in Ghana, church attendance is habitual and unremarkable; statements of faith and Biblical quotes are frequent on social media and everyday interactions; graduate students frequently offer their gratitude to God on their dissertations; and Uber, taxi, and bus drivers suspend religious icons from their rear-vision mirrors as a means of ensuring safe travel. While secularism is a feature of contemporary life in many high-income countries, it is not at all assumed in most of the world, certainly not across Africa.

Ian Whitmarsh and Elizabeth Roberts (2016, p. 207) suggest that modern medicine makes invisible the religious affects and sensibilities of the modern political/biological individual, that is, through its discourse of scientific truths and technical practices, biomedicine appears to constitute the human as *secular*, a knowable, reducible, biological entity. But in her ethnographic article, Roberts (2016) makes clear that the *non-secular*—faith and its enactment—*enables* the work of modern medicine. In her work in Ecuador, she describes how practitioners in assisted reproduction—clinical directors, embryologists, and fertility specialists—participate in “a specific form of religiosity, and they lived in relationship with petri dishes, chemicals, IVF patients, and God” (2016, p. 209–210). The divine and the human, technical knowledge and objects, large and particulate, all contribute to the work of assisted reproduction, such that “when I found petri dishes and God in labs, God stayed center stage” (2016, p. 211). As we illustrate below, this is true also in Ghana and South Africa.

## Results

Major findings from the study are presented below organized by themes that emerged from the interviews and observations (see Table 2).

**Table 2:** Summary of major findings

Country	Findings
Ghana	There was a focus upon religion in one of the two clinics in which fieldwork was conducted, various practices such as preaching to patients, communal prayers, and religious singing were routine and created sacred space within the clinic Emphasis placed upon the ultimate authority of divine power to determine the outcomes of fertility treatments Surrogacy remains a religious dilemma for some patients, along with concerns about witchcraft Staff openly declared their beliefs and undertook prayers and rituals to assist their scientific and care work. Religious beliefs were seen to assist in guiding moral clinical practice
South Africa	Religion was openly evident in one clinic where praying, Christian messages and references to the Bible were evident, another clinic specialized in providing services religiously acceptable to Jewish patients; other clinics were more secular in their presentation but recognized the varied religious and moral concerns of some patients undertaking IVF procedures Practices such as ova and sperm donation pose religious dilemmas for some patients, with particular implications for people whose cultures included beliefs in the importance of ancestral patrilineages Staff may not openly declare their beliefs but many did use prayer and other rituals to assist their scientific and care work

### Sacred Space in the Clinic, the Clinic as Sacred Space

Some clinics display their religious affiliations prominently; others appear to be strictly secular and biomedical. For example, in Ghana, one of the clinics explicitly profiled itself as religiously inspired: the landing page of the clinic’s website includes comments that the clinic was established “by the Grace of God, who brings hope and salvation through his Son Jesus Christ”. At the entrance to the building, visitors are welcomed with the words "Soli Deo Gloria", Latin for "Glory to God alone". A poster on the door leading to the IVF laboratory—which all clinic visitors pass—shows the belly of a pregnant woman, presumably referring to the result of a successful scientific IVF treatment, yet the poster bears the text "God will do it for you". Bibles can be found all over the clinic, including in the waiting room, on the desk of the clinic director, and in the wards.

Religious expressions were all pervasive and strongly visible in this clinic. Every morning, around 10 a.m., the clinic director, a gynaecologist who undertook his specialist training in IVF in Europe but is also a pastor, preaches to the women and men sitting in the waiting room. Chairs are positioned as if in a church setting, and for around an hour, the pastor/director leads joint prayers and appeals to God for success in resolving couples’ difficulties conceiving, and promises submission to His will. Women in the ward rooms were often found reading the Bible or some other religious book, or they would listen to religious music; early in the morning they began the day with a communal prayer and singing.

During ward rounds, the doctor gave women encouraging words, referring to "the Grace of God" to provide them with hope and confidence. Occasionally, he advised them to use a particular Bible verse in their prayers. Before the embryo transfer, a crucial step in IVF, clinic staff, and the woman in treatment prayed together.

During one interview with one of the nurse counsellors, Gerrits was suddenly interrupted by one of the gynaecologists, who told them about the successful delivery of twins by one of the surrogates. The nurse immediately took her mobile phone out of her pocket and phoned the intended parents. When they answered, instead of the nurse announcing the birth of the twins, she started to sing—with a beautiful voice—a song about the greatness of the Lord. Such obvious expressions of religiosity were not as evident at the other clinic which Gerrits studied in Ghana.

Most of our field sites in South Africa did not have overtly visible religious displays. Public hospitals appeared uniformly secular except for one public clinic which had a Bible available in the waiting room. The private clinics we visited had personal touches, like photographs by the doctors, or a book of photographs of the infants of couples who had had successful IVF treatment, but little religious imagery. One private clinic had a collection of fertility dolls from across the country on display; this appeared less as a gesture of devotion than as a display of curios for the benefit of the many foreign patients who visit that clinic.

However, one private clinic was overt about their Christian faith. This clinic had Bibles available in the drawers next to the bed in the waiting ward. In that clinic, the Christian faith of staff clearly infused their practice. For example, Nurse Neleka commented: "We are not against it [the singing of hymns], we are actually more positive to it, we are not scared to say God’s name… because all of us here are religious." Nevertheless, some expressions of Christian faith were not practiced for practical reasons:

I know many of the hospitals sing [hymns] before on-duty time, but because transport is always the issue, and then they [the staff] first want to sing, and they don’t sing one song only… and then they sing and then they sing two or three songs … you are so inspired and … and then you lose contact [lose track of time] and you miss the doctors’ rounds and then the patient must go to theatre, or this one then wants to deliver [a baby] but you are singing. So, we do not do that. We do respect everybody’s religion here, we do not force anything on anyone.

Prayer was the most common manifestation of Christian religious faith that staff spoke about in both the Ghanaian and South African sites. The sounds of prayer, hymns being sung, or religious music being played, create a sacred space within clinics. So too did the silent moments of reflection and pauses to express a quiet prayer before undertaking a procedure. In the South African clinic described above, Nurse Neleka spoke of colleagues who would hold hands and pray with patients who wished such support. Prayers were offered not only to make treatment failures more acceptable (as we will further discuss below), but to give women hope and encouragement. Many women in the Ghanaian clinic highly appreciated the daily preachings and shared prayer with the gynaecologist, in the hope that God would give them strength and help them achieve their goal.

### "I am Only a channel that God is Using"

Although not every clinician or embryologist referred to their faith in our interviews, many made reference to small quotidian acts of devotion through which they asked for divine support for some of the crucial tasks in IVF, such as egg retrievals, fertilization, and embryo transfers. For example, Dr Mandisa, working in an apparently secular private clinic in South Africa stated: “I always pray. I always pray when I am going to do an embryo transfer. I always pray. I take a moment of silence to say, ‘it is out of my hands, it is in Your hands God’. And then…[mimed releasing an embryo]”. Dr Unathi in South Africa described herself as:

Unapologetically Christian so the clients that come to me, they know that they are coming to see a Christian doctor and it is okay if you feel uncomfortable. Obviously, you will ask them before but …I definitely will pray before [procedures], my belief is that my success doesn’t just come from me but it comes from God, and I can only succeed if I invite God into the operation or whatever procedure I do. And it keeps me humble, that it is actually not my success, it is actually God that wills it and it makes the clients also comfortable that I’m not God. Especially with the … reproductive techniques. You cannot guarantee them that they are going to give us success and this is only when the two of us understand that I can only do up to how far I have been trained... I will do everything that I can do. But despite all that, between me and the couple, we can only go so far, the rest is up to God.

Similarly, several embryologists stated they offer a short prayer over fertilized ova and embryos, or have a particular small ritual they undertake. For example, although working in an apparently secular clinic, embryologist Anje stated:

I’ve got this imaginary idea that there’s a hole in the roof where my prayers go up because you know sometimes you sit there and especially when you’re dealing with testicular biopsy. There are minimal, minimal sperm and there are lots and lots of debris and you need to find that sperm and you’ve got eggs that are waiting. So I pray. I believe in the power of prayer, and I just ask God continually to bless my eyes, to bless my hands, and you know, you will not believe it, but the moment you’ve prayed that then you see a sperm … (Laughing). So God is good. God is good always.

Aditya Bharadwaj (2006, p. 458) sees such engagement with divine forces as a way to help “the doctor to manage the questions that biomedicine leaves unanswered”. One of the major unanswered questions in IVF, Bharadwaj adds, is why so many embryo transfers lead to treatment failures. One of the embryologists working in the Ghanaian clinic articulated it as follows:

We all know that sperms and eggs are not created by embryologists or doctors. We are only the vessels, bringing things together. I am only a channel that God is using to bring another baby into the world. They should not see the embryologist and medical doctors as God. We cannot manufacture eggs and sperms. What happens in the womb, we do not know!

These quotes not only reflect the uncertainties and unknowns operating in conception through IVF that staff—and patients—confront on a daily basis, but also an acknowledgement of their dependence on God to achieve this. The same view was expressed on the poster text on a door leading into the IVF laboratory in the Ghanaian clinic, indicating "God will do it for you"—clinic staff state clearly that to achieve their scientific results; they ultimately depend on the will of God. During the daily preaching and prayer for staff and patients, the infertility doctor in the Ghanaian clinic insisted: “When we successfully solve these problems, it is not the work of doctors or nurses or other paramedical practitioners. No, it is all the work of God! … God is the creator of life!”.

Only by acknowledging the superiority of God in the creation of life—assisted conception involves God’s helping hand acting through and with technology (Thompson, 2006, p. 559)—ARTs can be offered and used in this Ghanaian clinic. Hiadzi and colleagues (2021, p. 9) also suggest that for this reason, their study participants never blamed the staff for treatment failures. When treatment failed, according to their respondents, God was “never seen as opposed to their desire to have children” (2021, p. 9). Rather, it was not yet “the right time” for them. And for some people the right time may never come, a pastor explained, referring to examples of women in the Bible who remained childless: “everyone has a purpose on this earth (…) God’s plan for such people is different” (2021, p.10).

It is during moments of greatest strain and emotion, such as embryo transfers and surgical interventions, when patients seek assistance and solace in the divine. As the poster text on the door leading into the IVF laboratory in Ghana indicated—"God will do it for you"—clinic staff state clearly that to achieve their scientific results, they ultimately depend on the will of God.

### Guidance to Ethical and Moral Clinical Practice

Personal religious faith was also described as a guide to ethical practice. For example, Nurse Nekela in the visibly religious South African private clinic mentioned that due to her faith, she would not be involved or assist with abortions, and explained that “I am not very fond of the idea of two mothers or two fathers, because it's not natural”. Similarly, Akhona and Elsabe, ova donor coordinators in a different and not overtly Christian South African clinic, describe the partly similar dilemmas they face as practicing Christians, including the provision of treatment to gay couples or non-believers:

So one question that somebody asked the other time, they said to me, "You are a Christian, how do you manage to help for example gay guys or people that don’t believe at all, or people that believe in whatever?" … We all have different beliefs and it works for us and it is not my job to judge whoever believes what, I will not question them as much as they are not questioning me. So we have that respect for people and we help whoever in the same sense. I have my belief, everybody else out there is also allowed to have their own belief. So ja.

We pray in the morning, we set our day right, we ask God to just guide us because we are dealing with different people and different backgrounds, and we know that everything is in His hands, He is in control at the end of the day. And it is not our place to judge or put any assumptions on them and treat people differently. So we really just bring it back to Him so that we can do our best because He has placed us here for a reason and a time, and we just want to respect that and do our best.

In her response to the person questioning her about how she—as a Christian—acted in this field of ARTs, Akhona espouses an ethos of respect, tolerance and compassion for a diversity of religious beliefs and people and—by extension—the treatment of gay people is not to be questioned. Her answer shows, once again, her subordination to God: “Everything we do we are doing for the glory of God and whatever we do we must do with all our hearts”.

In Ghana too, when talking about the way his "religion" informed clinical practices, the embryologist referred to the staff’s duty to perform ARTs in an ethical and morally correct, as well as technically correct way. This included not discarding embryos: “I am realizing that it is another child that we do away with this. I feel guilty.” This moral position—not wanting to discard embryos—may have inspired the practice of egg sharing, which was common in the clinic. Egg sharing here refers to the practice that ova produced by one egg donor are used—or shared—by several couples to avoid freezing or discarding surplus embryos.

Clinicians and medical staff also spoke of their need to be aware of and attempt to modify their practice in order to cater for the religious beliefs of their patients. One clinic we visited is notable for its care for treating members of the South African Jewish population. This clinic not only had to deal with strict requirements to ensure what was jokingly referred to as "Kosher IVF", but the small size of the religious community in South Africa means confidentiality is crucial. Dr Michael has a Rabbi oversee his practices to ensure Rabbinical laws are observed. This includes having observers watch the embryologist at work to ensure no "mixing" of gametes accidentally occurs (Kahn, 2000). He noted that at first his embryologists found this confronting, implying that they weren’t good at their jobs, but they later came to accept the necessity of this if they were to work in their patients’ best interests. He explained:

[It is] very difficult, there are many laws involved and many restrictions involved… but there are [Jewish] couples that do use donors. They are not allowed to use Jewish donors, so it has to be a non-Jewish donor, and again there are permissions that are needed and it is a very difficult process for them, and that is not spoken about at all. It would be very rare if they disclose and if they tell… It would be an enormous secret and enormous shame, which I think is a very tough process because you carry this burden which should not be a burden all the time.

### Dilemmas of translation

Technologies such as IVF—which we view as socio-technological entities—travel to different cultural and religious contexts, and in this context, undergo a process of translation whereby they gradually become accepted components of reproductive repertoires (Hadolt et al., 2012; Inhorn & Birenbaum-Carmeli, 2008). Evidence of this process was provided by Akhona (34 years old) and Elsabe (30 years old), two ova donor coordinators at a clinic in Cape Town. In a discussion of cultures and religions, they made the following observations:

Akhona: There is somebody [a donor] who did not want to be chosen [by a patient] in their culture and I asked them why and she said, "because in the Zulu culture when a child is born we bring them back to the ancestors, so I do not want that to happen because that will be the wrong ancestors…."

Yes. So I remember sitting with this one patient and they had the same concern. They said, "Obviously we are going to give our child back to the ancestors"—when the child is back they do a proper ceremony, a clan name, they speak clan names, so she was saying "what clan names am I going to speak to this child?"

And I said to them…. we are fertilizing with your husband’s sperm. That is your husband’s clan names. So if we are not talking about your clan names, at least bring your child back to his clan name. You can bring the child’s ceremony back to the husband–which is most common in the African culture, the father is the only person, the father is the important part, the mother just carries the child. So I always say, if you are using your husband’s sperm then you don’t need to worry about that because it is going to be his clan name anyway.

In this exchange of views during a spontaneous discussion in an interview, the two women pondered how best to incorporate third-party donation into ancestor beliefs. As Ainslie (2014) describes, veneration of ancestors remains an important activity for many Zulu. Emphasis is primarily on the patrilineage. Men are considered the *intsika/entloko yekhaya* (literally the pillar/head of the home), and slaughter rituals for ancestor veneration remain essential to good fortune and health. Maintaining relationships and paying respect *hlonipha* to the ancestors is important for preventing illness and for the functioning of the family. If a new child is born and the ancestors do not recognize the child as part of their lineage, the child will be vulnerable to illness and misfortune due to witchcraft. Questions of how to explain children born through third-party donation to the ancestors, and concerns by donors that any child born will not be introduced to the "right" ancestors (and hence she and the child will suffer the consequences), are all issues that need to be navigated.

Third-party sperm donation poses particular difficulties as it involves the use of non-patrilineal sperm, and we were informed it is generally not accepted by infertile African men. In the case of severe male infertility, intracytoplasmic sperm injection (ICSI) is preferred, involving the injection of a single sperm into each egg to assist fertilization. However, the existence of a sperm bank in Cape Town suggests that the lower cost practice of third-party sperm donation is practiced in some cases, albeit with great discretion. With no central authority ruling on these matters, such as happens with a *fatwa* ruling in Islam or a pontificate statement for Roman Catholics, individuals are left to ponder these dilemmas.

Similarly, Gerrits encountered a surrogate in Ghana who had decided to become a surrogate to earn money to be able to better care for her children and was deeply concerned about the ethics of her actions. After the embryo transfer, her worries increased and she started to have sleep problems and nightmares. Her worries included thoughts about whether this surrogacy practice was “spiritually correct” or “sinful”, and whether witchcraft could be involved:

The eggs that are in me may come from a witch person who has a bad spirit or even the sperm. … if the egg and sperm come [in contact] with the blood. I am thinking if you can pass (on) the witchcraft?

She had shared her concerns with the clinic doctors, who had tried to encourage her and advised her to pray. “When we are in sin”, the doctor had said, “we can always ask forgiveness”. The doctor had pointed her to reflect upon one particular biblical verse which reflects on fear (1 John 4:18).^2^ Two and a half months after the embryo transfer, she said the prayer had consoled her and given her peace of mind.

Witchcraft accusations remain important across Sub-Saharan Africa, marking transgressions of the social order and explaining a range of unforetold or undesirable events, including in relation to health and reproduction. Women who have failed to have children are often targets of such accusations, in some cases leading to violence and expulsion from their villages (Abebe et al., 2020; Gerrits et al., 2023; Hiadzi et al., 2021; Ofosu-Budu & Hanninen, 2020). It is understandable that a woman who is ambiguous about the social acceptability and ethics of her actions might consider whether witchcraft is involved. The doctor both acknowledges her ontological realities—informed by ideas on witchcraft—and he offers a religious solution—contemplating a bible verse "makes treatment possible" (Thompson, 2006, p. 557).

## Limitations

A longer in-depth ethnography would reveal more of the ways in which IVF is incorporated into the social beliefs and practices around reproduction. Our data were limited to that offered spontaneously by our sample of informants, and there is need for further study of the views of patients and their experiences of religious practice within clinic encounters. The clinics we visited had little to say about Islamic practices, although some clinics in South Africa and Ghana provided care to Muslim patients from countries like Nigeria and Tanzania.

## Conclusions

In her ethnography in Ecuador, Elizabeth Roberts (2012, p. 8) notes that in many clinics where IVF ethnographies have been conducted, participants situate IVF within a framework where reproduction is seen as primarily a biological process. Such clinics assert the autonomy of the woman, de-emphasizing the wider relational factors involved in family-making. However, in Ecuador, she argues, IVF is situated within the ontologies of local people, for whom reproduction never occurs without assistance from God and larger family relations. Similarly, in Thailand, appeals to the magic and sacred remain commonplace among patients and doctors as they partake in high-tech IVF (Whittaker, 2015). Likewise, as summarized in Table 2, in Ghana and South Africa, IVF is situated within a context in which the mystery of reproduction and limits of scientific knowledge and biomedicine remain acknowledged; for these informants, all reproduction is assisted by a higher force. Small spiritual acts and prayers remain common to the management of health risks and concerns in these settings, as for all things—success at school, a new car, smooth work relations.

We explored the creation of sacred spaces in clinics. Not all clinics actively practice spiritual interventions alongside assisted reproduction, but one of the Ghanaian clinics publicly promotes itself as doing so, thereby appealing to particular groups of patients. In South Africa, religious practice was less public. Nevertheless, in these clinics appeals to the divine were evident through the smaller personal rituals and prayers of some staff members. Such appeals not only acknowledge the limitations of IVF technologies; they may also explain its failures and successes, as these are to a large extent in the hands of God—staff constantly repeat that they are “only a channel that God is using.” These appeals position staff and patients as moral subjects who have done everything possible to address the vagaries of assisted reproduction—another form of care to enable, complement and enhance high-tech intervention.

Quests for children in these contexts by necessity involve consideration of social, spiritual, and religious implications, tribal allegiances, and questions of personal faith. They also involve consideration of the future implications for kin and patrilineage of the creation of a new child. The implications of these new technologies are still being thought through and are not yet socially settled. For example, how will ancestors react to third-party assistance? Can witchcraft be transmitted through sperm and eggs? This is a sign that IVF reproduction is not entirely integrated into the reproductive repertoires of some people in these societies. In a commentary on ARTs and religion, Linda Layne (2006) suggests a list of other contentious IVF issues which require further research in various faith settings. These include the status of embryos and understandings of the beginning of life; name giving practices before birth; whether selective reduction of embryos is allowed; and practices around the disposal of unused embryos and aborted embryos.

The outcomes of technological processes of assisted reproduction are doubtful and often seem to hinge upon factors other than hormone protocols, laboratory care, or the age of the patient. In IVF treatment, the chances of failure outweigh those of success, and hence doctors and patients talk about the role of destiny and divine intervention in their treatment. Given that the outcomes of processes like IVF appear God-given, there is good reason that the babies that result are described as "miracles".

## Footnotes

1 African Zionism is a religious movement with 15–18 million members throughout Southern Africa combining Christianity and African traditional religion.

2 There is no fear in love. But perfect love drives out fear, because fear has to do with punishment. The one who fears is not made perfect in love. (1 John 4:18).
